# Machine Learning and Radiogenomics: Lessons Learned and Future Directions

**DOI:** 10.3389/fonc.2018.00228

**Published:** 2018-06-21

**Authors:** John Kang, Tiziana Rancati, Sangkyu Lee, Jung Hun Oh, Sarah L. Kerns, Jacob G. Scott, Russell Schwartz, Seyoung Kim, Barry S. Rosenstein

**Affiliations:** ^1^Department of Radiation Oncology, University of Rochester Medical Center, Rochester, NY, United States; ^2^Prostate Cancer Program, Fondazione IRCCS Istituto Nazionale dei Tumori, Milan, Italy; ^3^Department of Medical Physics, Memorial Sloan Kettering Cancer Center, New York, NY, United States; ^4^Department of Translational Hematology and Oncology Research, Cleveland Clinic, Cleveland, OH, United States; ^5^Department of Radiation Oncology, Cleveland Clinic, Cleveland, OH, United States; ^6^Computational Biology Department, Carnegie Mellon School of Computer Science, Pittsburgh, PA, United States; ^7^Department of Biological Sciences, Carnegie Mellon University, Pittsburgh, PA, United States; ^8^Department of Radiation Oncology, Icahn School of Medicine at Mount Sinai, New York, NY, United States; ^9^Department of Genetics and Genomic Sciences, Icahn School of Medicine at Mount Sinai, New York, NY, United States

**Keywords:** statistical genetics and genomics, radiation oncology, computational genomics, precision oncology, machine learning in radiation oncology, big data, predictive modeling

## Abstract

Due to the rapid increase in the availability of patient data, there is significant interest in precision medicine that could facilitate the development of a personalized treatment plan for each patient on an individual basis. Radiation oncology is particularly suited for predictive machine learning (ML) models due to the enormous amount of diagnostic data used as input and therapeutic data generated as output. An emerging field in precision radiation oncology that can take advantage of ML approaches is radiogenomics, which is the study of the impact of genomic variations on the sensitivity of normal and tumor tissue to radiation. Currently, patients undergoing radiotherapy are treated using uniform dose constraints specific to the tumor and surrounding normal tissues. This is suboptimal in many ways. First, the dose that can be delivered to the target volume may be insufficient for control but is constrained by the surrounding normal tissue, as dose escalation can lead to significant morbidity and rare. Second, two patients with nearly identical dose distributions can have substantially different acute and late toxicities, resulting in lengthy treatment breaks and suboptimal control, or chronic morbidities leading to poor quality of life. Despite significant advances in radiogenomics, the magnitude of the genetic contribution to radiation response far exceeds our current understanding of individual risk variants. In the field of genomics, ML methods are being used to extract harder-to-detect knowledge, but these methods have yet to fully penetrate radiogenomics. Hence, the goal of this publication is to provide an overview of ML as it applies to radiogenomics. We begin with a brief history of radiogenomics and its relationship to precision medicine. We then introduce ML and compare it to statistical hypothesis testing to reflect on shared lessons and to avoid common pitfalls. Current ML approaches to genome-wide association studies are examined. The application of ML specifically to radiogenomics is next presented. We end with important lessons for the proper integration of ML into radiogenomics.

## 1 Introduction to Radiogenomics

### 1.1 Normal Tissue Toxicity Directly Limits Tumor Control

Over 50 years before the discovery of the DNA double helix, radiation therapy and normal tissue radiobiology became irrevocably linked after Antoine Henri Becquerel left a container of radium in his vest pocket, causing a burn-like reaction of erythema followed by ulceration and necrosis (1, 2). Ever since, the goal of therapeutic radiation has been to deliver a maximal effective dose while minimizing toxicity to normal tissues. The importance of this goal has increased as cancers that were previously fatal became curable and patients have had to live with long-lasting late effects and secondary malignancies (3, 4).

For several tumors, an argument can be made that survival is so poor that one should not be as concerned for late effects. However, acute toxicity may also constrain dose escalation, which directly limits tumor control, since a therapeutically efficacious dose may not be achievable due to toxicity. This is because dose tolerances are typically set for 5–10% toxicity in clinical trials, so the patients with the most radiosensitive normal tissue ultimately determine the limit for the maximum dosage for all patients (5, 6). As Becquerel noted, tumor control and normal tissue toxicity have been, and remain, irrevocably linked. Advances in the last decades from the fields of radiation physics and radiation biology have focused on finding ways to separate these two effects with varying success, as discussed below.

### 1.2 Technology Has Improved Normal Tissue Toxicity

To improve therapeutic ratio (i.e., the cost–benefit of tumor control vs. normal tissue side effects) in recent decades, medical physics has made significant advances in the technology and techniques of radiation delivery to spare normal tissue (7). This includes moving from 2D treatment planning using X-ray films to 3D planning using CT-simulation, and now to inverse planning and fluence modulation to create conformal dose distributions employing intensity-modulated radiation therapy (IMRT) (8). IMRT not only utilizes more sophisticated hardware but also advanced treatment planning software and optimization algorithms. Multiple prospective and retrospective studies have demonstrated the superiority of IMRT in reducing toxicity for most solid cancer types, including those of the head and neck (9), lung (10), prostate (11), anus (8), and soft tissue sarcoma (12). Utilizing protons for cancer treatment provides another way to increase dose conformality and decrease normal tissue dose through the Bragg peak. Complementary technologies include improvements in image guidance (13), motion management (14), and patient positioning (15). Radiosurgery for central nervous system tumors is an attractive alternative to lengthier and more toxic treatments. Brachytherapy also offers dosimetric advantages to decrease toxicity and improve tumor control. Due to the successes of the technological advancements, there has been relatively fast adoption of emerging physics technologies in the clinic as standard of care in many places.

### 1.3 Radiobiology and Normal Tissue Toxicity

While radiation physics was using increasingly complex methods and data to perform more individualized treatments, advancements in radiation biology were also developing, but have yet to achieve the same level of clinical impact. Early efforts in the 1980s and 1990s to employ radiation biology approaches in the clinic focused on altered fractionation schedules to improve control of head and neck tumors and small cell lung cancer while sparing normal tissue toxicity. These trials demonstrated benefits to both hyperfractionation (16, 17) and accelerated fractionation (18, 19), but these protocols have not translated into changes in the standard of care at many centers or into similar studies in most cancers (20). Therapies for modulating tissue oxygenation and the use of hypoxic cell radiosensitizers and bioreductive drugs have been moderately successful in animal studies and randomized clinical trials (21) but also have not yet reached wide penetration in the United States despite level I evidence, often due to side effects. More recently, hypofractionation (i.e., larger doses of radiation per fraction) has become widely adopted; however, there is significant controversy as to how this can best be modeled (22–26). Whereas advances in radiation physics brought about measurable improvements in both tumor control and normal tissue protection as demonstrated through multiple clinical trials—largely due to IMRT—this could not be said for advances in radiobiology. It became clear that a different approach other than modeling of fractionation would be necessary to keep pace with the increasing torrent of clinical data. Such an opportunity would arise at the turn of the twenty-first century with substantial advances in molecular biology and the first draft of the human genome (27, 28) as discussed below.

### 1.4 Genomic Basis for Radiotherapy Response

Through studies of patients following radiotherapy (29, 30), it has become apparent that patient-related characteristics, including genomic factors, could influence susceptibility for the development of radiation-related toxicities (31). To identify the genomic factors that may be associated with normal tissue toxicities, a series of candidate gene studies was performed that resulted in more than 100 publications from 1997 to 2015 (32). However, with a few exceptions, the findings were largely inconclusive, and independent validations were rare (33). The risk of spurious single-nucleotide polymorphism (SNP) associations has been a concern for candidate gene association studies even before the advent of genome-wide association studies (GWAS) (34).

With improved understanding of the genetic architecture of complex traits, we now know that a few variants in limited pathways—such as DNA damage response—cannot alone explain most of variation in radiotherapy response. While this work was in progress, results of the Human Genome Project and related efforts demonstrated the magnitude of genetic variation between individuals. Over 90% of this variation comes from common SNPs (frequency >1%) and rare variants. There are about 10 million common SNPs in the human genome and any locus can be affected. These variants can be in coding regions (exons), introns, or intergenic regulatory regions. Early efforts to understand how SNPs were linked to phenotypic traits were marred by poor statistical understanding of correction for multiple hypothesis testing, which led to multiple small and underpowered studies (35).

To improve power to detect new SNP biomarkers for radiation toxicity, the International Radiogenomics Consortium (RGC) was formed in 2009 to pool individual cohorts and research groups. One of the main goals is to determine germline predisposition to radiation toxicity and there have been several studies from RGC investigators that have identified novel risk SNPs.

REQUITE is a project led by RGC members to prospectively collect clinical and biological data, and genetic information for 5,300 lung, prostate, and breast cancer patients (36). The RGC also collaborates with the GAME-ON oncoarray initiative (32).

#### 1.4.1 Fundamental Hypothesis of Radiogenomics

Andreassen et al. reported three basic hypotheses of radiogenomics (32):

(a) Normal tissue radiosensitivity is as a complex trait dependent on the combined influence of sequence alteration of several genes.
(b) SNPs may make up a proportion of the genetics underlying differences in clinical normal tissue radiosensitivity.
(c) Some genetic alterations are expressed selectively through certain types of normal tissue reactions, whereas others exhibit a “global” impact on radiosensitivity.

Regarding these hypotheses, it is prudent to add that we are now aware that there are also epigenetic components of normal tissue radiosensitivity that are—by definition—not captured by genetic sequences but are heritable nonetheless.

#### 1.4.2 The Importance of Fishing

Genome-wide association studies could certainly be categorized as a “fishing expedition,” which has pejorative connotations given the history of improper correction for multiple hypothesis testing (see Multiple Hypothesis Correction). However, fishing expeditions in genomics are a necessity to generate new hypotheses. Recent GWAS performed by members of the RGC have been able to identify novel associations of SNPs in genes that were previously not linked with radiation toxicity (37). For example, *TANC1* is a gene that encodes a repair protein for muscle damage and is one such example of a novel radiosensitivity association discovered in 2014 (38). A meta-analysis of four GWAS also identified two SNPs, rs17599026 in *KDM3B* and rs27720298 in *DNAH5*, which are associated with increased urinary frequency and decreased urinary stream, respectively (39).

### 1.5 Precision Medicine and Single Drug Targets

Compared to biomarker panels for normal tissue toxicity to radiation therapy, the realm of biomarker panels for prediction of tumor response is a much wider field, as it also encompasses the domains of medical and surgical oncology. Early successes in predictive biomarkers focused on single mutations, such as the *BCR-ABL* translocation observed in chronic lymphocytic leukemia or oncogene amplification, such as *Her2-neu* or *EGFR*. In the last half decade, therapies targeting tyrosine kinase mutations in lung cancer or high expressing immune markers in many tissue types have become standard of care. In March 2017, the US Food and Drug Administration (FDA) granted a tissue-agnostic “blanket approval” for the PD-1 inhibitor pembrolizumab for any metastatic or unresectable solid tumor with specific mismatch repair mutations (40); this was the first time FDA approval had been granted for a specific mutation regardless of tumor type.

Given the various targeted agents, there are many who herald this as the age of “precision medicine.” In late 2016, the American Society for Clinical Oncology (ASCO) launched Journal of Clinical Oncology (JCO) subjournals “JCO Clinical Cancer Informatics” and “JCO Precision Oncology.” In accordance with the single target–single drug approach, contemporary precision medicine drug trials are based on amassing targetable single mutations (NCI-MATCH) or pathway mutations (NCI-MPACT) (41). While the initial tumor response can be quite impressive, durable response is an issue as single-target drugs are prone to develop resistance (42, 43).

### 1.6 Precision Medicine and Multigene Panels

Since the discovery of the Philadelphia chromosome and imantinib, most drugs remain focused on single biomarkers, such as a single mutation or a gene expression alteration with a large penetrance. However, we are rapidly depleting the pool of undiscovered, highly penetrant genes. Soon, targeting the low hanging fruit through a one gene–one phenotype approach will no longer be sufficient for effective “precision medicine.” This is where multiple biomarker panels are making an impact. While these do not necessarily provide “multiple targets” for drugs to act on, they do provide a prognostic picture of the effects of tumor mutational burden. The earliest and most well known of these laboratory-developed biomarker panels are the 21-gene recurrence score Oncotype DX (Genomic Health, Inc., Redwood City, CA, USA) (44) and 70-gene MammaPrint (Agendia BV, The Netherlands) (45). These panels are used to make critical clinical decisions regarding whether select breast cancer patients are predicted to benefit from chemotherapy.

Current efforts are aimed at understanding the genomic signature of metastatic cancer. Memorial Sloan Kettering has used their MSK-IMPACT gene expression panel to sequence tumors from over 10,000 patients with metastatic disease to be able to prognosticate whether a future patient will develop metastases (46). While the development of these laboratory tests requires significant investment, they may ultimately save substantial sums by decreasing unnecessary therapies and toxicities while improving quality of life for cancer patients.

Recent discussions about the state of precision medicine and genomically guided radiation therapy include a review by Baumann et al. (7) and a joint report by the American Society for Radiation Oncology (ASTRO), American Association of Physicists in Medicine (AAPM), and National Cancer Institute (NCI) summarizing a 2016 precision medicine symposium (6) (see Promoting Research).

A complicating factor in tumor genomics is a result of tumor heterogeneity, which results in different subtypes within the same tumor, as shown in glioblastoma (47), colorectal cancer (48), and pancreatic cancer (49). Given the limited ability of single-target drugs, therapies may select certain subclones of higher fitness to predominate and create mechanisms of resistance. Selection occurs not only from therapy but also from local and microenvironment constraints (50), leading to an increasingly robust evolutionary model of tumor heterogeneity obeying Darwinian selection. Distant metastases display this evolutionary behavior as well as they seed further distant metastases (51). To better target a tumor’s genomic landscape, we may need to sample multiple spatially separated sites and incorporate evolutionary analysis (52).

### 1.7 Tumor Control and Radiogenomics

Although a substantial emphasis of radiogenomics has been to identify biomarkers predictive of normal tissue toxicities, there are efforts being made to develop tests for tumor response to radiation (53). In the largest preclinical study, Yard et al. showed that there is a rich diversity of resultant mutations after exposing 533 cell lines across 26 tumor types to radiation (54). Within these tumor cell lines, radiation *sensitivity* was enriched in gene sets associated with DNA damage response, cell cycle, chromatin organization, and RNA metabolism. By contrast, radiation *resistance* was associated with cellular signaling, lipid metabolism and transport, stem-cell fate, cellular stress, and inflammation.

PORTOS is a 24-gene biomarker predictive assay that can determine which post-prostatectomy patients would benefit from post-operative radiation therapy to decrease their 10-year distant metastasis-free survival (55). PORTOS is the first of future clinical radiogenomics assays to help determine which patients will benefit from radiation.

The radiosensitivity index (RSI) was developed at Moffitt Cancer Center to predict radiation sensitivity in multiple tumor types (56, 57). Its signature is based on linear regression on the expression of 10 specific genes (*AR, cJun, STAT1, PKC, RelA, cABL, SUOMO1, CDK1, HDAC1*, and *IRF1*) that were chosen from a pool of over >7,000 genes using a pruning method derived from systems biology principles. These genes are implicated in pathways involved in DNA damage response, histone deacetylation, cell cycle, apoptosis, and proliferation. More recently, the RSI has been combined with the linear quadratic model of cell kill to create a unified model of both radiobiologic and genomic variables to predict for radiation response and provide a quantitative link from genomics to clinical dosing (58).

## 2 Introduction to Machine Learning (ML)

Machine learning is a field evolved from computer science, artificial intelligence, and statistical inference that seeks to uncover patterns in data to make future predictions. Unlike handcrafted heuristic models often seen in clinical medicine, ML methods have a foundation in statistical theory and are generalizable to a type of problem as opposed to specific problems (59). There are many ML methods, and each has unique advantages and disadvantages that merit consideration by the user prior to attempting to model their results (60, 61). Similarly, there are several ML-friendly programming languages and specialized libraries to choose from, including Python’s Scikit-learn package (62), MATLAB’s Statistics and Machine Learning Toolbox (63), and R (64).

### 2.1 Statistical Inference vs. ML

Machine learning has considerable overlap with classical statistics and many key principles and methods were developed by statisticians. There continues to be considerable crossover between computer science and statistics. Breiman wrote about the differences between the two fields, calling ML the field of black box “algorithmic models” and statistics the field of inferential “data models” (65).

In ML, models are commonly validated by various measures of raw predictive performance, whereas in statistics, models are evaluated by goodness of fit to a presumptive model. These models can be used for either explaining or predicting phenomena (66). One key difference that readers of clinical papers will immediately notice is that formal hypothesis testing is a rarity in ML. This stems from the fact that ML is concerned with using prior information to improve models, rather than inferring a “belief” between two hypotheses. Classical hypothesis testing—used in most clinical studies—relies on the frequentist approach to probability. In this interpretation, one selects a level of belief α and—assuming a certain probability distribution—then determines whether the obtained result is extreme enough such that if the experiment was repeated many times, one would see this result at a rate of ≤α. This rate is called the *p*-value, and the significance level α is typically set at 0.05. ML papers rarely discuss significance levels, instead seeking to identify maximum likelihood models or sample over spaces of possible models, as in Bayesian statistics. To determine significance levels requires some assumptions regarding the distribution implied by a null hypothesis for the data, which is more difficult for complex problems such as speech recognition, image recognition, and recommender systems.

### 2.2 An Update of Breiman’s Lessons From ML

In 2001, Breiman noted three important lessons from ML over the prior 5 years: the Rashomon effect, the Occam dilemma, and the curse of dimensionality. Here, we will re-visit these to discuss relevance to contemporary issues of ML usage in medicine.

#### 2.2.1 Rashomon Effect

The Rashomon effect describes a multiplicity of models where there are many “crowded” models that have very similar performance (i.e., accuracies within 0.01) but which may have very different compositions (i.e., different input variables). Within oncology, this effect is well demonstrated in breast cancer where Fan et al. showed that four of five different gene expression models (including MammaPrint and Oncotype DX Recurrence Score) showed significant agreement in patient prognosis despite having very different inputs (67). This model crowding is magnified by variable pruning (i.e., feature selection) as the remaining variables must then *implicitly* carry the effect of the removed variables. The Rashomon effect is popularly seen in nutritional epidemiology where observational studies routinely seem to show conflicting data about the risk or benefits of certain supplements (68). This phenomenon was studied in Vitamin E, where depending on which combinations of 13 covariates were selected, one could find a range in increase or decrease of Vitamin E-associated mortality—a so-called “vibration of effects” (69).

The Rashomon effect can manifest as model instability when multiple Monte Carlo repetitions of cross-validated model selection are performed (see CV Methodology) that result in different models selected in each repetition. This occurs due to minor perturbations in the data resulting from different splits and is particularly magnified for smaller datasets. Ensemble models (70) and regularization methods (see Embedding Feature Selection With the Prediction Model) (71) seem to work well for addressing this problem.

#### 2.2.2 Occam Dilemma

William of Occam (c. 1285–1349) described the Principle of Parsimony as: “one should not increase, beyond what is necessary, the number of entities required to explain anything.” Breiman describes the Occam dilemma as the choice between simplicity—and interpretability—and accuracy. He noted that simple classifiers—such as decision trees and logistic regression (LR)—were interpretable but were easily outclassed in classification performance by more complex and less-interpretable classifiers like random forests (RFs). However, increasing model complexity also tends to overfit. This dilemma has been partially mitigated by a better understanding of cross validation (CV) (see Cross Validation) as well as better strategies for automated control for model complexity.

**Figure 1:**
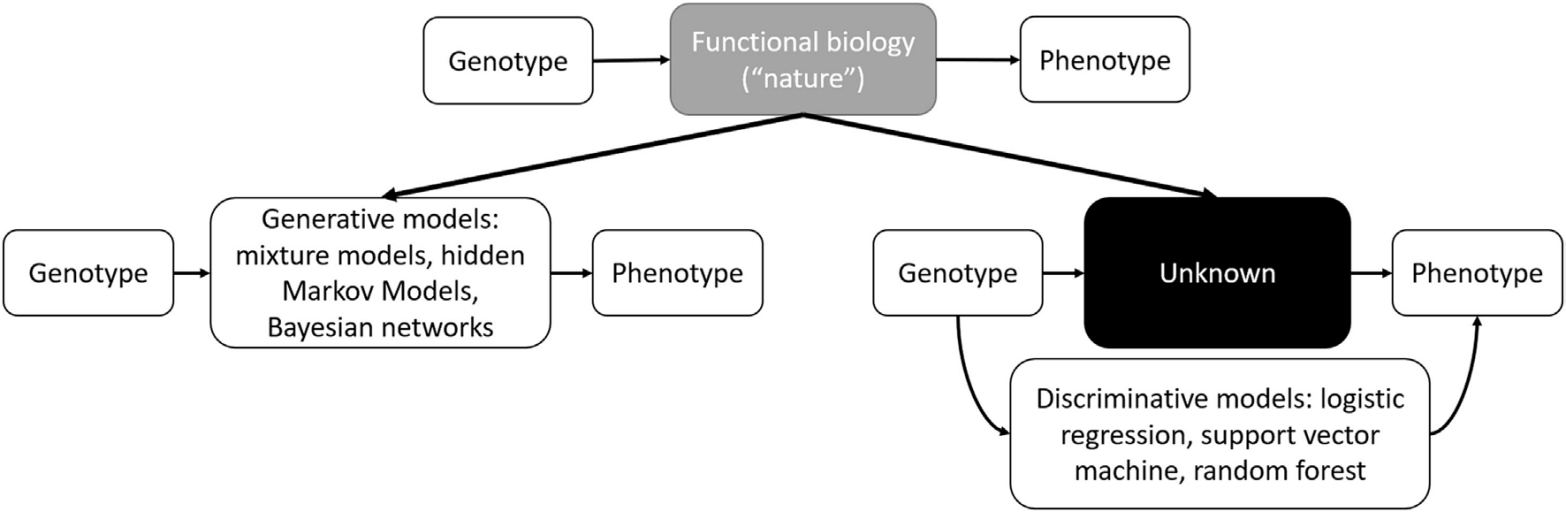
Schematic outline of functional biology modeling *via* generative or discriminative models.

In contemporary usage, where the boundary between interpretable statistical models and “black box” ML models has become blurred, interpretability and accuracy discussions have resurfaced in the form of generative and discriminative models. Generative approaches resemble statistical models where the full joint distribution of features is modeled (see Bayesian Networks). Discriminative approaches focus on optimizing classification accuracy using conditional distributions to separating classes (see Support Vector Machines). Both of these approaches have been described in ML applications to genomics (72). Generative models are more interpretable and handle missing data better, whereas discriminative classifiers perform better asymptotically with larger datasets (73). Thus, we can update Breiman’s interpretation with a contemporary interpretation of modeling genetic information (Figure 1).

Breiman had postulated that physicians would reject less-interpretable models, but this has not been the case. As discussed in Section “Precision Medicine and Multigene Panels,” oncology is moving toward validating and using high-dimensional multigene models in the clinic to guide treatment decisions.

As a future where a multigene panel for all cancers is still a long way off, creating intuitive models is still relevant. Patients can rarely be placed into neat boxes, and physicians must often incorporate clinical experience, which becomes more difficult for less-interpretable models. A method that was developed to overcome this limitation is MediBoost, which attempts to emulate the performance of RF while maintaining the intuition of classic decision trees (74). In Section “Current ML Approaches to Radiogenomics,” we discuss the interpretability of three ML methods.

#### 2.2.3 The Curse of Dimensionality

The curse of dimensionality refers to the phenomenon where potential data space increases exponentially with the number of dimensions (75). For example, a cluster of points on a line of length 3 au appears much more desolate when clustered in a cube of volume 27 au^3^. Two things happen with increasing dimensions: (1) available data becomes increasingly sparse and (2) the number of possible solutions increases exponentially while each can become statistically insignificant by overfitting to noise (76). Traditional thinking has always been to try to reduce feature number; however, some ML methods benefit from higher dimensions. For example, when data are nearly linearly separable, LR and linear support vector machine (SVM) perform similarly. However, when data are *not* linearly separable, SVM can use the kernel trick that increases the dimensionality of data to allow separation in higher dimension (see Support Vector Machines). While SVM has built-in protections for this “curse” by defining kernel functions around the data points themselves and selecting only the most important support vectors, it remains vulnerable when too many support vectors are selected with high-dimensional kernels.

Within genomics, the curse of dimensionality is reflected in the difficulty of finding epistatic interactions (77). In standard search for additive genetic variance, one needs to only search *n* SNPs in a single dimension. However, if pairwise or higher-order interactions are considered, then the search space increases exponentially; for example, the search space for pairwise interactions is *n*(*n* − 1)/2. Traversing the large but sparse search space while maintaining reasonable performance can be a challenge (see Combining ML and Hypothesis Testing).

#### 2.2.4 ML Workflow

**Figure 2:**
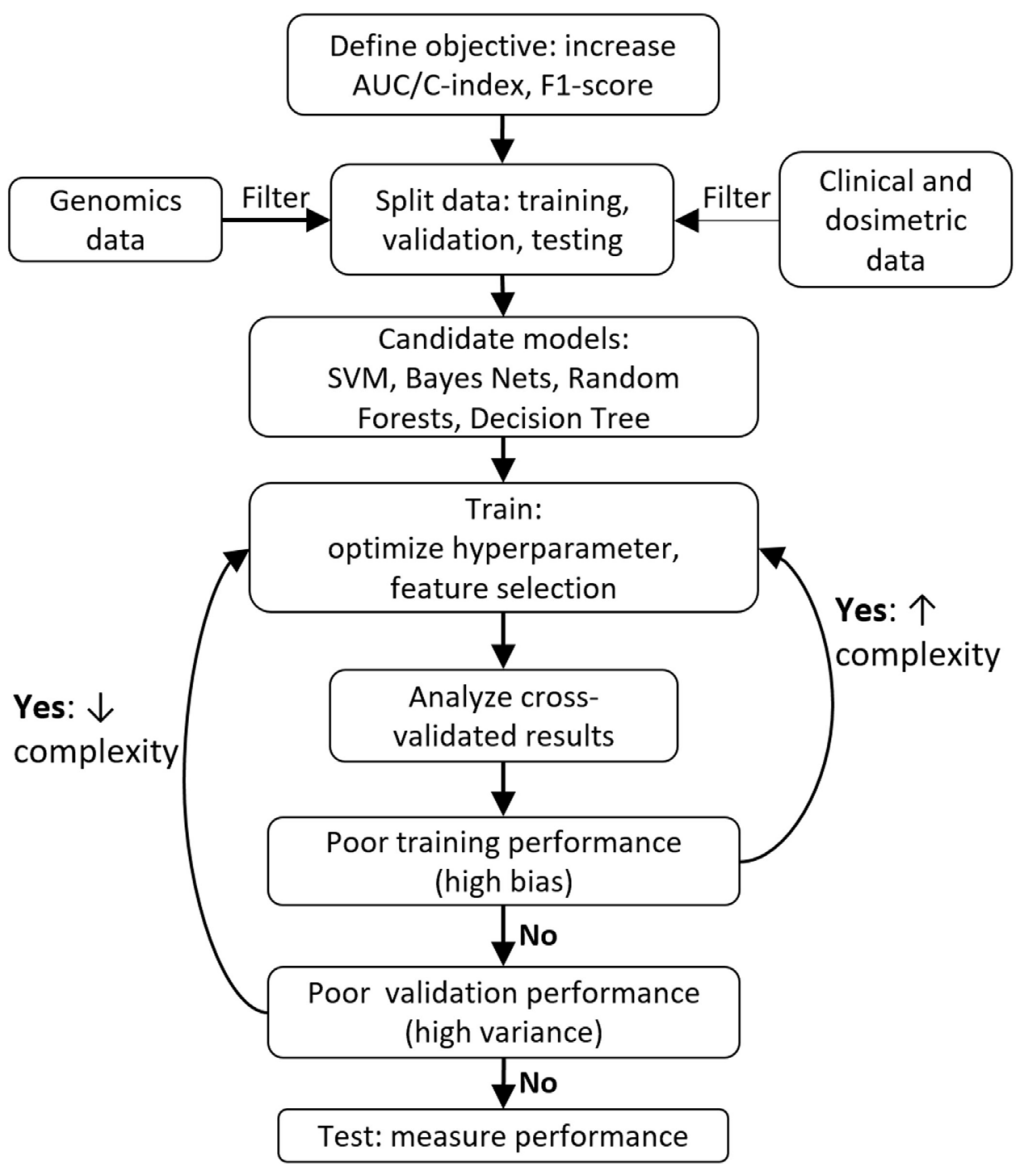
Typical machine learning project workflow.

In an ideal world, there would exist a perfect protocol to follow that will guarantee a great ML model every time. Unfortunately, there is no consensus on the “optimal” way to create a model. Libbrecht and Noble described general guidelines for applying ML to genomics (72). Within radiation oncology, Lambin et al. provide a high-level overview of clinical decision support systems (78). Kang et al. discussed general ML design principles with case examples of radiotherapy toxicity prediction (60). El Naqa et al. provide a comprehensive textbook of ML in radiation oncology and medical physics (79). Figure 2 provides a sample workflow for a general radiation oncology project that incorporates both genomics and clinical/dosimetric data. Two critical components of model selection include “Cross validation” and “Feature selection,” which are further discussed below.

### 2.3 Cross Validation

The greater the number of parameters in a model, the better it will fit a given set of data. As datasets have become more and more complex, there has become an inherent bias toward increasing the number of parameters. Overfitting describes the phenomenon of creating an overly complex model which may fit a given data set, but will fail to generalize (i.e., fit another data set sampled from a similar population). CV is a method used in model selection aimed to prevent overfitting by estimating how well a model will generalize to unseen data.

#### 2.3.1 CV Methodology

Conceptually, CV is used to prevent overfitting by training with data separate from validation data. As an example, in *k*-fold CV (KF-CV) for *k* = 10, the data are initially divided into 10 equal parts. Next, 9 parts are used to train a model while the 10th part is used to assess for how well the model was trained in the validation step. This training–validation procedure is run 9 more times, with each of the 10 parts taking turns as the validation set. The performance averaged over 10 runs is the cross-validated estimate of how well the model will perform on truly unseen data. The optimal number of initial splits for the data has not been established, but 10-fold CV is commonly used. An alternative to KF-CV that is often used for smaller datasets is “leave one out” cross-validation (LOO-CV), whereby a dataset of size *n* is split into *n* parts. This form of CV maximizes the relative amount of information used for training the model while minimizing the information used for testing. As a result, LOO-CV is prone to higher variance (i.e., a higher propensity to overfit) and decreased bias (i.e., a lower propensity to underfit) compared with KF-CV. Similar to balancing type I and type II error in statistical genetics, variance and bias must be carefully considered to avoid “false positive” and “false negative” results.

#### 2.3.2 CV Relationship With Statistical Inference

Cross validation took some time to catch on in statistics literature, but has long been a fundamental part of the algorithmic ML models (65). Due to the lack of interpretability in the “black box,” ML has relied on CV and related methods like bootstrapping to demonstrate robust performance without relying formally on statistical significance. Small sample sizes can be a problem for creating prediction models. In this case, learning curve analysis can be used to create empirical scaling models, whereby one varies the size of the training set to assess for learning rate (80). Learning curve analysis can be used to help determine at what point a model is overfitting (81). When learning curve analysis predicts large error rates that are unlikely to be significant, permutation testing can predict the significance of a classifier by comparing its performance with that of random classifiers trained on randomly permuted data (80).

### 2.4 Common Errors in CV

When performed correctly, CV is a powerful tool for selecting models that will generalize to new data. However, this seemingly simple technique is infamous for being used incorrectly. This creates an especially egregious problem as using CV gives results an appearance of rigorous methodology when the exact opposite may be occurring.

#### 2.4.1 Violating the Independence Assumption

A common mistake is to pre-maturely “show” the test data while still training the model and thus violate the independence assumption between the training and test data. For example, a typical workflow is to set aside test data and train a model using only the training data. Once the training results are acceptable, the model is tested on the independent testing data. If the testing results are unacceptable, one might then use these results to refine the model. However, using performance on the test set to guide decisions for training, the model creates bias and violates the independence assumption between the model design and testing (82). The more repetitions of model pruning are performed, the higher the chance of the model overfitting to truly independent data. See Section “Reusable Hold-Out Set” for a solution to this problem.

Sometimes, re-using training samples in testing is intentional. This was the case in the MammaPrint assay, where the authors used a large proportion of the tumor samples from the initial discovery study in their validation study (83, 84). The authors claimed this was necessary due to an imbalance of tumor cases and controls (see Section “Unbalanced Datasets” below for solutions).

In part due to the lack of independence between the testing and training sets in biomedical research, which culminated in the pre-mature use of omics-based tests used in cancer clinical trials at Duke University (85, 86), the Institute of Medicine released a report in 2012 (84). Several cautionary steps were advised, including validating with a blinded dataset from another institution (see Replication and Regulatory Concerns).

#### 2.4.2 Freedman’s Paradox

Freedman showed that in high-dimensional data, some variables will be randomly associated with an outcome variable by chance alone and if these are selected out in model selection, they will appear to be strongly significant in an effect called Freedman’s paradox (87). This can occur even with no relationship between the input variables and outcome variables because with enough input variables, by chance one will have a high correlation. Even if model selection is performed and low performing variables are removed, the same randomly associated features will remain correlated and appear to be highly significant. Freedman’s paradox manifests when CV is repeated to perform both model selection and performance estimation. One solution is to use cross model validation, also known as nested CV: the outer loop is used for performance estimation and the inner loop for model selection (88–90).

### 2.5 Feature Selection

Often, one is interested in not only fitting an optimal model but rather in determining which of the variables—also known as features—are the most “important” through the process of feature selection. With respect to ML in genomics, Libbrecht and Noble described three ways to define “importance” in feature selection (72). The first is to identify a very small subset of features that still has excellent performance (i.e., to create a cheaper SNP array to test association with a phenotype rather than whole genome sequencing). The second is to attempt to understand underlying biology by determining which genes are the most relevant. The third is to improve predictive performance by removing redundant or noisy genes that only serve to overfit the model. The authors note, unfortunately, that it is usually very difficult to perform all three simultaneously.

There are two general methods for feature selection (and can be used together). One is using domain knowledge *via* feature engineering and one is utilizing automated approaches. In feature engineering, a domain expert may pick and choose variables from a larger pool that he or she thinks are important prior to more formal model selection. As discussed in Section “Rashomon Effect,” this bias can often lead to spurious conclusions when different research groups pre-select their variables (69). In many genomics applications, often precurated gene ontology data are referenced at some point through a hypothesis-driven approach, either as an initial screen or as part inferring functional relationships after significant genes have been selected. This does introduce a bias toward highly studied gene functions or pathways and a bias against undiscovered gene function, which reinforces the importance of hypothesis-generating studies (see The Importance of Fishing).

Below, we discuss automated approaches for feature selection. The first two are general approaches that are either pre-processing features through a method independent or dependent of the final predictive model. A third approach is to transform the existing features to create new synthetic features (91).

#### 2.5.1 Pre-Processing Variables Independent of the Prediction Model

Filtering (or ranking) variables is the least computationally intensive method for feature selection. This method involves selecting features prior to training a model and is thus independent of the model choice. A common method is to perform univariate correlation testing (for continuous variables) or receiver operating curve analysis (for categorical variables) and then only choosing the top-ranking variables. While efficient in that the processing time scales linearly with the number of variables, filtering does not screen out highly correlated features—in fact, these will be more likely to be selected together. However, Guyon and Elisseeff did show that presumably redundant variables can decrease noise and consequently improve classification (91). Statistically, filtering variables is robust against overfitting as it aims to reduce variance by introducing bias (92). Univariate filtering methods do not consider interactions between features, and thus is unable to assist in determining what variable combination is optimal. In GWAS, statistical tests for univariate significance are an example of variable filtering and thus are unable to account for multi-locus interactions (93). This weakness is magnified when a variable that is uninformative by itself gains value when combined with another variable, as is proposed in epistasis; in this case, filtering would remove the univariately useless variable before it can be tested in combination with another variable. To address this weakness, filter methods such as the ReliefF family take a multivariate and ensemble approach to yield variable rankings (94–96).

#### 2.5.2 Embedding Feature Selection With the Prediction Model

Combining feature selection with the model establishes a dependence that can be used to address issues with multicollinearity and feature interactions. Wrappers combine feature selection with model building but are computationally expensive (97). Various search strategies can be utilized, but often used are greedy search strategies where predictors are either added or removed one-by-one *via* forward selection or backward elimination, respectively. In regularization, feature selection is built into a method’s objective function (i.e., the optimization goal) through penalty parameters. These penalty parameters ensure that feature importance (weight) and/or number is incorporated during model training. Common regularization methods include L1-norm or lasso regression (98), L2-norm or ridge regression (99), and combined L1–L2 or elastic networks (100). Regularization methods are of significant interest in applications of ML to genomics due to their ability to decrease the complexity of a polygenic problem and improve probability of replication (90). A relatively novel method developed for feature selection in very high dimensions is stability selection, which uses subsampling along with a selection algorithm to select out important features (101).

#### 2.5.3 Feature Construction and Transformation

Instead of working directly with the given features, features can be manipulated to reconstruct the data in a better way or to improve predictive performance. There are many methods that can perform feature construction with different levels of complexity. Clustering is a classic and simple method for feature construction that replaces observed features by fewer features called cluster centroids (102). Principal component analysis (PCA) provides a method related to eigenvector analysis to create synthetic features which can explain the majority of the information in the data; for example, PCA can decrease type I error by uncovering linkage disequilibrium (LD) patterns in genome-wide analyses due to ancestry (103, 104). Kernel-based methods such as SVMs also make use of feature transformation into higher dimensions and will be discussed in a later Section “Support Vector Machines.” Neural networks are another popular ML method that specializes in constructing features within the hidden layers after being initialized with observed features. In the last few years, neural networks have become extremely popular in the form of deep learning, which is discussed below.

### 2.6 Deep Learning

“Deep learning” describes a class of neural networks that has exploded in popularity in the recent years—particularly in the fields of computer vision (105) and natural language processing (106)—as larger training data sets have become available and computational processing resources have become more accessible and affordable (107). Deep learning is distinguished from earlier neural network methods by its complexity: whereas a “shallow” neural network may have only a few hidden layers, deep learning networks may have dozens (108) to hundreds of layers (109) where unsupervised, hierarchical feature transformation can occur. In popular science, deep learning is the artificial intelligence powering IBM Watson (110) and autonomous driving vehicles. Within medical research, there have been several high-profile deep learning publications claiming expert-level diagnostic performance (111–114). A related domain is radiomics, which seeks to use ML and statistical methods to extract informative imaging features or “phenotypes” in medical imaging (115–117) with a significant focus in oncology imaging (118–121). Deep learning is in an early stage within genomics, but has been used for discovery of sites for regulation or splicing (122, 123), variant calling (124), and prediction of variant functions (125). For further reading on deep learning, we recommend Lecun et al.’s excellent review (107).

## 3 ML in Genomics

Genomics presents a challenging problem for ML as most methods were not originally developed for GWAS, and thus improving implementations remain a topic of ongoing research (126). The quantity of genomics data recommended for finding significant SNPs is more akin to that seen in image processing, where there could be tens of millions of voxels in a typical computed tomography scan. Given the imbalance of features compared with samples (the “p ≫ n” problem), there is a challenge in creating predictive models that do not overfit. As discussed in Section “Current ML Approaches to Radiogenomics,” different ML methods have been used to address different concerns in genomics and radiogenomics.

In this section, we will review some of the intuition and principles behind genomics methods to better understand how to improve and apply them to future problems.

### 3.1 Multiple Hypothesis Correction

Hypothesis testing is a principle based on statistical inference. In GWAS, however, one is not just testing a single hypothesis, but millions. As such, by random chance, it is a virtual guarantee that some of the associations will appear to be statistically significant if there is no correction to the pre-specified significance level α (127). How to correct for multiple hypothesis comparisons is an area of significant interest in GWAS and there are many techniques to do so (128). These methods generally aim to control the number of type I errors and include family-wise error rate (FWER)—the probability of at least one type I error—and false discovery rate—the expected proportion of false discoveries (129). Controlling FDR has greater power than FWER at the risk of increased type II error (130). One common FWER correction method is Bonferroni correction, which would work reasonably well for independent tests, but is an overly strict (i.e., conservative) bound for GWAS due to the prevalence of LD across the genome. LD causes adjacent regions of the genome to be inherited together, and thus Bonferroni will overcorrect due to non-independence among SNPs within LD blocks. For rare variants which are not thought to be in LD, Bonferroni correction would be an appropriate correction.

In ML, poor correction for multiple testing is related to p-hacking or data dredging, which is to continuously run iterations of this method until it fits a pre-conceived notion or hypothesis (131) (see Lessons From Statistics).

### 3.2 The Case of Missing Heritability

As sample sizes have increased since the first GWAS in 2005, more and more robust associations with loci have been discovered in genomics (132). This has also been reflected in radiogenomics as larger sample sizes have been possible through the RGC (see Genomic Basis for Radiotherapy Response). However, the discovered associations are still relatively few and insufficient to explain the range of observed phenotypes, creating the so-called “case of missing heritability” (133). Response of both normal and tumor tissue has certainly shown itself to be a complex, polygenic trait (29, 30, 54). The cause of this missing heritability is thought to arise from several sources, including common variants of low effect size, rare variants, epistasis, and environmental factors. One clear solution already underway is to genotype more samples and to use meta-analysis methods to combine results across studies (134). However, there are limits to this approach. For one, rare variants [minor allele frequency (MAF) < 0.0005] with smaller effect sizes (odds ratios ~1.2) will require between 1 and 10 million samples for detection using standard GWAS techniques (132). Another issue is that epistatic interactions among common variants have not been able to be reliably replicated (77). ML provides a complementary approach for finding patterns in noisy, complex data and detecting non-linear interactions.

### 3.3 Combining ML and Hypothesis Testing

Originally, two-stage GWAS was developed from standard one-stage GWAS to decrease genotyping costs in an era where SNP chips were costlier (135). In this method, all SNP markers are genotyped in a proportion of the samples in stage 1, and a subset of the SNPs would then be selected for follow-up in stage 2 on the remaining samples. This method does not decrease type I or II error, however (136). Performing a joint analysis where the test statistics in stage 2 were conditional on stage 1 had superior results than assuming independence between the two stages (i.e., a replication study), but power is unable to exceed that of one-stage GWAS (137). Instead of two-stage GWAS, a promising alternative is to use two-stage models combining ML and statistical hypothesis testing, aiming to combine the strengths of separate methodologies (see Statistical Inference vs. ML). These combined models can increase power and uncover epistatic interactions (138).

#### 3.3.1 Learning Curves and Power

**Figure 3:**
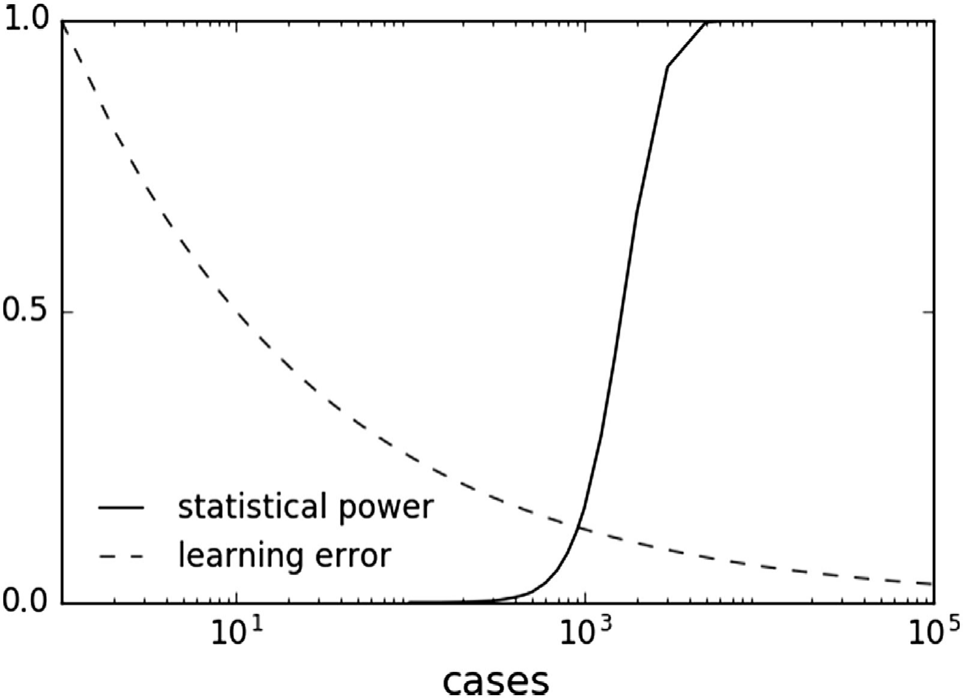
Sample plots of statistical power and learning curve error. Statistical power graph derived using Genomic Association Studies power calculator (137). Learning curve assuming an inverse power law common to multiple machine learning methods (80, 139, 140).

In principle, combining ML and hypothesis testing works because, by design with setting a pre-determined alpha level and power, statistical inference does not benefit from larger datasets once a result has met statistical significance. Indeed, larger datasets can result in detection of statistically significant associations of decreasing effect size and potentially decreasing clinical relevance. This limitation does not apply to ML, which can asymptotically use more data to improve predictive performance. Many ML methods are characterized by a learning rate obeying an inverse power law with respect to sample size (80, 139, 140). This behavior suggests that ML offers a complementary approach to statistical methods by continuing to learn for each additional sample. With increasing sample sizes and meta-analyses, one can imagine a scenario where one is well in the “plateau” portion of the power curve and can afford samples to be used in the ML method (Figure 3).

#### 3.3.2 Using ML to Detect Epistasis

Epistasis, which includes interactions between SNPs, is not well accounted for in standard GWAS. Epistatic interactions are recognized as a cause of non-linear effects and may help elucidate functional mechanisms as well (141). Biological interpretations of epistasis have been difficult with little correlation between statistical interaction and physical interaction (i.e., protein–protein binding) or other biologic interactions (142). Regardless of whether protein products are physically interacting with other proteins or environment, the statistical interaction suggests that there is dependence at some level for a specific disease (141).

Given the exponentially increased search space for SNP interactions, there is a high concern for false positives (see The Curse of Dimensionality). This concern is magnified when SNPs are in LD. A filtering method is often used to decrease the search space for only the most promising interactions (see Pre-Processing Variables Independent of the Prediction Model). Exhaustive searches for pairwise interactions are also now becoming possible, aided by the massive advances in parallel processing throughout offered by graphical processing units (143, 144).

Due to technical limitations in accounting for non-linear effects and multiple hypothesis correction in an exhaustive search, interaction studies have typically focused on SNPs with weak marginal effects (77). Unfortunately, many of the studies in non-cancer diseases have not been successful (145, 146). One postulate is that pairwise SNPs are unlikely to have large interaction effects. However, as sample sizes and SNP density improve (to better tag causal variants while avoiding spurious interactions due to LD), then ML methods that incorporate SNP interactions with low or no marginal/main effects may begin to uncover replicable interaction effects (138, 147–149).

Two-stage methods are a promising approach that combines the strength of fast, approximate interaction tests with a subsequent thorough model (77). Such methods take advantage of the strength of statistical tests for detecting polygenic low signal, linear interactions with the ability of ML to train cross-validated models of non-linear interactions (150, 151). Regularization within two-stage methods is an area of interest (90). Wu et al. adapted lasso to LR for use in dichotomous traits in GWAS (152). Wasserman and Roeder developed a similar procedure called “screen and clean” that also controls for type I error by combining lasso linear regression, cross-validated model selection, and hypothesis testing (153). Like traditional two-stage GWAS, the data are split between the stages. Wu et al. adopted this model to model interaction effects in addition to main effects (154).

As further discussed in Section “Random Forest,” ensemble tree-based methods are very popular for detection of epistatic interactions (148, 155, 156). While it is difficult to assess statistical significance in ensemble black box techniques, permutation re-sampling methods can be used to determine a null distribution and associated *p*-values (80, 138, 141) (see CV Relationship With Statistical Inference). Other popular methods for interaction that have continued to receive updates include a cross-validated dimensionality reduction method called multifactor dimensionality reduction (157) and a Markov Chain Monte Carlo sampling method to maximize posterior probability called Bayesian Epistasis Association Mapping (158).

#### 3.3.3 Using ML to Increase Power

Overfitting and false discoveries (type I errors) represent similar concepts in ML and statistical inference, respectively, in that both falsely ascribe importance. Like the bias-variance tradeoff, statistical inference seeks to balance type I and type II errors. As each hypothesis test represents an additional penalty to genome-wide significance, one way to decrease type II error is to decrease the number of hypothesis tests. While decreasing testable hypotheses may appear to decrease power, Skol et al. demonstrate that being more stringent in selecting SNPs in stage 1 may paradoxically increase power as the multiple testing penalty is subsequently reduced in stage 2 (137).

Combination of ML and statistical methods can simultaneously be designed to detect epistasis and increase power (138). In “screen and clean” (see Using ML to Detect Epistasis), Wasserman and Roeder perform L1-regularization in the “clean” phases to improve power in the “screen” phases. Meinshausen et al. extend the method by Wasserman and Roeder by performing multiple random splits (instead of one static split) to decrease false positives and increase power (159). Mieth et al. similarly combined SVM with hypothesis testing (160), but instead of splitting, they re-sample data using an FWER correction (161). While re-sampling for feature selection and parameter tuning may bias toward more optimistic results (see Freedman’s Paradox), Mieth et al. report higher power compared with Meinshausen and Wasserman and Roeder, with 80% of the discovered SNPs validated by prior studies. Nguyen et al. took a similar approach except with RF instead of SVM (162).

Combined ML and statistical methods can either have the ML stage first or second. When ML is used first, it usually acts as a feature selection filter to reduce the multiple hypothesis penalty and increase power for hypothesis testing in the second stage. When the ML step is second, it acts to validate candidate SNPs that passed the first stage filter. The order of ML and hypothesis testing may not affect power. Mieth et al. report similar results compared with Roshan et al. (163), who performed chi-square testing followed by RF or SVM [supplement in Ref. (160)]. Similarly, Shi et al. proposed single SNP hypothesis testing followed by lasso regression, which was the reverse order of Wasserman and Roeder (164).

Oh et al. used a multi-stage approach to uncover novel SNPs and improve prostate radiotherapy toxicity prediction (165, 166). The first step is to create latent (indirectly observed) variables through PCA. These “pre-conditioned” variables are fit using LR to the original outcomes. This serves to create “pre-conditioned” outcomes that are continuous in nature and provides estimate of radiotoxicity probability. These pre-conditioned outcomes are then modeled using RF regression and validated on holdouts of the original samples.

## 4 Current ML Approaches to Radiogenomics

Machine learning models are particularly attractive when dealing with genetic information, as they can consider SNP–SNP interactions, which are suspected to be important, but are often missed by classical association tests because their marginal effects are too small to pass stringent genome-wide significance thresholds.

However, ML models also come with constitutional pitfalls, namely, increased computational complexity and risk for overfitting, which must be acknowledged and understood to avoid reporting impractical models or over-optimistic results.

Current use of ML techniques in radiogenomics usually follows the top-down approach, where radiotherapy outcomes are modeled through complex statistical analysis, without considering *a priori* knowledge of interactions of radiation with tissue and biological systems. In this field, supervised learning is widely preferred, i.e., models aim at constructing a genotype–phenotype relationship by learning such genetic patterns from a labeled set of training examples. Supervised learning can provide phenotypic predictions in new cases with similar genetic background. Nevertheless, an unsupervised approach (e.g., PCA or clustering) is sometimes used to reduce the dimensionality of datasets, extract a subset of relevant features, or construct features to be later included in the chosen learning method. Feature selection is of extreme importance (see Feature Selection), as it leads to the reduction of the dimensionality of the genetic search space, excluding correlated variants without independent contribution to the classification, and helping the translation of the model to the clinical setting.

Even if most ML techniques can act both as regression and classification methods, the classification or discriminative aspect has been most investigated in recent years, with main interest in separation between patients with/without the selected study outcome (e.g., presence/absence of radiotherapy-induced toxicity, tumor control/failure, and presence/absence of distant metastasis).

There is also increasing interest in overcoming the “black box” characteristics of some ML methods, favoring use of techniques that allow ready interpretation of their output (see Occam Dilemma), making apparent to the final user the relationships between variables and the size and directionality of their effect, i.e., if the variables are increasing or decreasing the probability of the outcome and the magnitude of their impact.

In this frame, RF, SVMs, and Bayesian networks (BNs) received great attention and they constitute the main topic of this section (Table 1). The presented ML algorithms can accommodate GWAS-level data. When considering the emerging sequencing domain (e.g., whole-exome and genome profiling), new technical challenges are posed that might be addressed by new algorithmic advances or by parallelization and cloud technologies for distributed memory and high-performance computing.

**Table 1:** Three representative machine learning methods with select pre-processing tips and tuning methods for complexity control.

Method	Pre-process	Complexity control	Reference
Support vector machine (SVM)	– Encode features as binary – Normalize to uniform distribution – Imputation for balancing data	– Recursive feature elimination for linear SVM – Soft margin width (C-parameter) – Kernel hyperparameters	(76, 160)

Bayesian networks	– Feature discretization – Variable selection to reduce graph search space – Imputation not necessary when using expectation maximization	– Constraints to a graph search space based on prior knowledge – Graph scoring functions that penalize complexity	(167–171)

Random forest	– No discretization or normalization necessary – Imputation required	– Number of features to sample at each node split (mtry) – Minimum number of samples in a terminal node	(172, 173)

### 4.1 Random Forest

Random forest is a regression and classification method based on an ensemble of decision trees (172). The ensemble approach averages the predicted values from individual trees to make a final prediction, thus sacrificing the interpretability of standard decision trees for increased prediction accuracy (74). Each tree is trained on bootstrapped training samples (i.e., sampling with replacement), while a random subset of features is used at each node split. When applied to a problem of predicting a disease state using SNPs, for example, each tree in the forest grows with a set of rules to divide the training samples based on discrete values of the genotypes (e.g., homozygous vs. heterozygous). Here, we list the characteristics of RF that make it an attractive choice for GWAS, both for outcome prediction and hypothesis generation.

#### 4.1.1 Robustness at High-Dimensional Data

Given high-dimensional data, training predictive models likely faces risk of overfitting. The ensemble approach utilized by RF mitigates this risk by reducing model variance due to aggregation of trees with low correlation. Examples of studies emphasizing predictive performance of RF include work by Cosgun et al. (174), Nguyen et al. (162), Oh et al. (165) (SNP based), Wu et al. (175), Díaz-Uriarte and Alvarez de Andrés (176), and Boulesteix et al. (177) (microarray based). While RF was initially thought not to overfit based on datasets from the UCI ML repository (65), this was ultimately found to be incorrect when noisier datasets were introduced (178). When training RF models, some parameters need to be optimized, which can affect predictive power. Among those, the number of variables that are randomly selected from the original set of variables at each node split (*mtry)* governs model complexity. Many studies opt for default configurations as originally recommended by Breiman (172) (classification: $\sqrt{p}$, regression: *p*/3 where *p*: number of predictors), and predictive performance was shown to be stable around these values (176, 179). However, a larger *mtry* is recommended when there are many weak predictors (172), which might be the case for GWAS of complex diseases. Goldstein et al. (173) conducted a search for optimal parameters in GWAS of multiple sclerosis, comprising about 300K SNPs, and recommended *mtry* = 0.1 after initial pruning of the SNPs under high LD.

#### 4.1.2 Biomarker Prioritization

Random forest can provide a variable importance measure (VIM), which quantifies the influence of an individual predictor on the purity of the node split (purity based) or prediction accuracy in unseen samples (permutation based). VIM can be used for selecting a smaller subset of genes or SNPs from GWAS, which can be further used for achieving higher predictive performance or biological validation. Lunetta et al. (180) proposed to use RF VIM for SNP prioritization as an alternative to Fisher’s *p*-value under the presence of SNP–SNP interactions. Nguyen et al. (162) used VIM as a feature selection process for a subsequent RF training to enhance predictive performance. However, reliability of VIM, especially under LD, has been questioned and investigated by simulation studies: Tolosi and Lengauer (181) and Nicodemus et al. (182) suggested that VIM may not correctly measure the importance of a large group of correlated SNPs due to dilution of VIM. Also, Strobl et al. (183) showed potential bias in VIM toward the predictors with more categories; they proposed the conditional inference tree as an alternative where each node split is performed based on a conditional independence test instead of the conventional Gini index (184).

#### 4.1.3 Ability to Account for SNP–SNP Interactions

Epistasis describes the non-linear combination of SNPs (or SNP and environment) that may correlate with a phenotype. Epistasis is thus important for understanding complex diseases (77). By construction, RF can indirectly account for epistasis through successive node splits in a tree where one node split is conditional upon the split from the previous node. Lunetta et al. (180) claimed that RF VIM has a higher power of detecting interacting SNPs than univariate tests. Thus, RF has been used as a screening step to identify much smaller number of SNPs that are more likely to demonstrate epistasis, which can be further tested in a pairwise fashion (150, 151). However, Winham et al. (156) warned that ability of RF VIM to detect interactions might decrease with an increasing number of SNPs and large MAF of SNPs.

#### 4.1.4 Hybrid Methods

Random forest is occasionally used in conjunction with other ML methods. Boulesteix et al. (177) used partial least squares to reduce dimensionality of gene microarray data prior to training a RF classifier. Stephan et al. (185) used RF as a fixed component of a mixed-effect model to handle population structure. Oh et al. (165) introduced a pre-conditioning step prior to RF training where a binary outcome of radiotherapy toxicity was converted to a continuous pre-conditioned target, which helps reduce the noise level that may be present in the outcome measurements (186).

### 4.2 Support Vector Machines

Support vector machines are usually used to solve the problem of supervised binary classification. In the field of oncologic modeling, SVMs are used to classify new patients into two separate classes (with/without the outcome of interest) based on their characteristics (76). The first step is to find an efficient boundary between patients with/without the outcome in the training set. This boundary is called a “soft margin” and is a function of the known *d* features of the patients included in the training set. To determine this boundary, non-linear SVMs use a technique called the kernel trick to transform data into a higher dimension, whereby they can then be separated by a *d-*dimensional surface in a non-linear fashion. Based on these transformations, SVM finds an optimal boundary between the possible outcomes. In technical terms, a linear SVM models the feature space (the space of possible support vectors, which is a finite-dimensional vector space where each dimension represents a feature) and creates a linear partition of the feature space by establishing a hyperplane separating the two possible outcomes. Of note, the created partition is linear in the vector space, but it can use the kernel trick to solve non-linear partition problems in the original space. Based on the characteristics of a new patient, the SVM model places the new subject above or below the separation hyperplane, leading to his/her categorization (with/without the clinical outcome). SVMs maximize the distance between the two outcome classes and allow for a defined number of cases to be on the “wrong side” of the boundary (i.e., a soft margin). Due to this, despite the complexity of the problem, the SVM boundary is only minimally influenced by outliers that are difficult to separate.

Support vector machines are a non-probabilistic classifier: the characteristics of the new patients fully control their location in the feature space, without involvement of stochastic elements. If a probabilistic interpretation for group classification is needed, the measure of the distance between the new patient and the decision boundary can be suggested as a potential metric to measure the effectiveness of the classification (187).

#### 4.2.1 Robustness in High-Dimensional Data and Possibility to Handle for Variable Interaction

Support vector machines are particularly suited to model datasets including genomic information, as they are tailored to predict the target outcome (the phenotype) from high-dimensional data (the genotype) with a possible complex and unknown correlation structure by means of adaptable non-linear classification boundaries. The framework of SVMs implicitly includes higher-order interactions between variables without having to predefine what they are. Examples of studies highlighting good performance of SVMs in this area are (188–190).

The main pitfall presents when the number of variables for each patient exceeds the number of patients in the training dataset. For this reason, in such case, the combination of SVMs with techniques aimed at reduction of the number of features is suggested.

Support vector machines can be used to approach analysis of GWAS data even in combination steps. Mieth et al. (160) proposed a two-step SVM procedure with SVMs first adopted for testing SNPs by taking their correlation structure into account and for determining a subset of relevant candidate SNPs (see Combining ML and Hypothesis Testing). Subsequently, statistical hypothesis testing is performed with an adequate threshold correction. As complexity reduction is performed prior to hypothesis testing, the strict multiple correction threshold can thus be relaxed.

#### 4.2.2 Tuning Parameters

Considering practical challenges in SVM modeling, a key issue is tuning the parameters identifying the separation hyperplane and determining how many support vectors must be used for classification. There are also kernel-specific parameters to tune. Grid search is traditionally used to find the best set, with choice of initial conditions and search strategy highly influencing the quality of the result (191, 192).

#### 4.2.3 Unbalanced Datasets

Attention must also be paid when SVMs are applied to unbalanced data, i.e., one outcome class contains considerably more cases than the other. This scenario is common in radiotherapy modeling where toxicity and local failure rates can be low. Unbalanced datasets present a challenge when training every type of classifier, but particularly is true for maximum-margin classifiers such as SVM. A satisfactory choice for having a high-accuracy classifier on a very imbalanced dataset could be to classify every patient as belonging to the majority class. Nevertheless, such a classifier is not very useful. The central issue is that, in such a case, the standard notion of accuracy is a bad measure of the success of a classifier, and a balanced success rate should be used in training the model, which assigns different costs for misclassification in each class (170, 193, 194). These methods can include showing a full confusion matrix; reporting F1-score and positive/negative predictive values, which incorporate relative imbalances (195–197); or synthetic balancing through undersampling and/or oversampling (198).

#### 4.2.4 Interpretation of SVMs

Interpreting SVM models is far from obvious. Consequently, work is being done in providing methods to visualize SMV results as nomograms to support interpretability (199, 200).

The absence of a direct probabilistic interpretation also makes SVM inference difficult, with the aforementioned work by Platt being one solution (187).

### 4.3 Bayesian Networks

Bayesian network is a graphical method to model joint probabilistic relationships among a set of random variables, meaning that the variables vary in some random or unexplained manner (201). Based on the analysis of input data or from expert opinion, the BN assigns probability factors to the various results. Once trained on a suitable dataset, the BN can be used to make predictions on new data not included in the training dataset.

A key feature of BN is graphical representation of the relationships *via* a directed acyclic graph (DAG). Although visualizing the structure of a BN is optional, it is a helpful way to understand the model. A DAG is made up of *nodes* (representing variables) and directed *links* between them, i.e., links originate from a parent variable and are pointed to child variables without backwards looping or two-way interactions. Parent variables influence the probability of child variables and the probability of each random variable is established to be conditional upon its parent variable(s). In this way, the DAG encodes the presence and direction of influence between variables, which makes BN attractive for users needing intuitive interpretation of results (169) (see Occam Dilemma). This directionality of links is important as it defines a unique representation for the multiplicative partitioning of the joint probability: the absence of an edge between two nodes indicates conditional independence of involved variables.

#### 4.3.1 Interpretation of BNs

**Figure 4:**
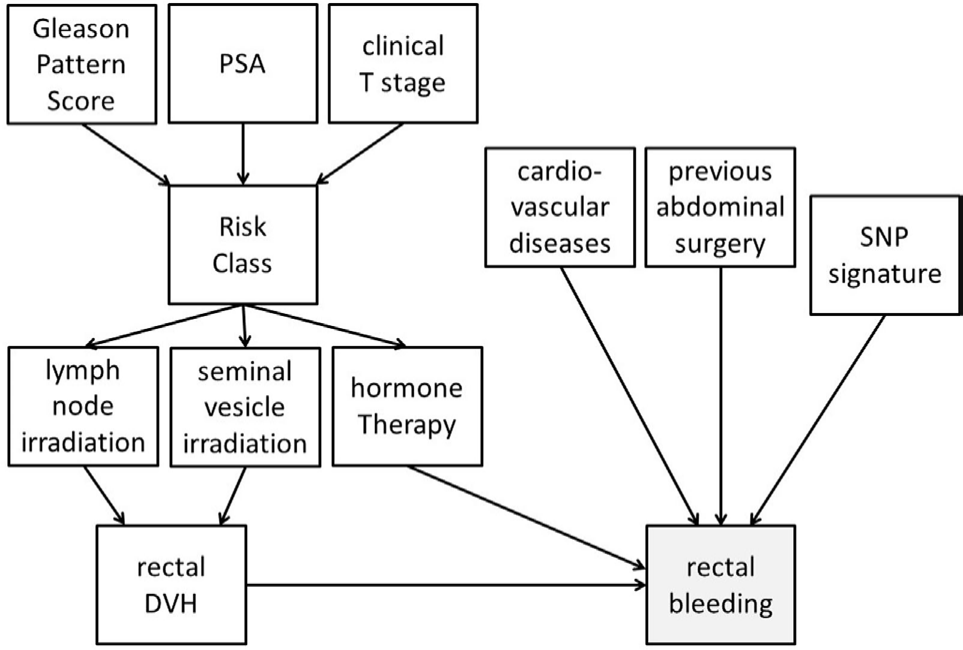
Possible representation of a Bayesian network directed acyclic graph for predicting late rectal bleeding after radiotherapy for prostate cancer. The network includes tumor-related characteristics (PSA, Gleason pattern score, and clinical T stage) which determine risk class and consequently radiotherapy targets (irradiation of pelvic lymph nodes and of seminal vesicles) and use of concomitant hormone therapy. Treatment variables influence the dosimetry of organs at risk [rectal dose–volume histogram (DVH)], and this has a causal effect on late rectal bleeding probability. Clinical (presence of a previous abdominal surgery and of cardiovascular diseases) and genetic [single-nucleotide polymorphism (SNP) signature] variables with (causal) associations with rectal bleeding are also included in the DAG.

Bayesian networks can integrate different data types into analysis. Despite accounting for high-order variable interactions (e.g., genetic environment), BNs maintain high interpretability *via* graphical outputs. As an example, Figure 4 demonstrates a possible BN for prediction of radiotherapy-induced rectal bleeding following different clinical, genetic, and treatment-related variables.

#### 4.3.2 Using Knowledge and Data in a Synergistic Way

A DAG can be built starting from previous knowledge, or completely trained on available data. For example, BN was used to incorporate expert knowledge along with experimental assay data to assign functional labels to yeast genes (202). The optimized DAG is the one which maximizes a predefined scoring function over all possible DAG configurations. When multiple DAGs score at the same level, an approach embracing an ensemble of models can be followed (169).

#### 4.3.3 Robustness at High-Dimensional Data

Since the number of possible DAGs grows super-exponentially with the number of available features, it is unrealistic to comprehensively search for the highest-scoring DAG over all graph possibilities. This is especially true when considering high-dimensionality problems encountered in GWAS. Various approaches could be suggested to confront the burden (169, 170):

(a) Use a causality prior that considers the already available knowledge to impose restrictions on the presence/direction of links between nodes to reduce the search space.
(b) Structure features into systems of different hierarchical levels with connections established by combining data and prior knowledge.
(c) Reduce input dimension by appropriate variable selection techniques with the aim of removing highly correlated features.
(d) Use of graph scoring functions that penalize complex graph structures, such as Bayesian information criteria (167).

An interesting approach is also the use of a forest of hierarchical latent class models (171) to reduce the dimension of the data to be further submitted to BN to discover genetic factors potentially involved in oncologic outcomes. Latent variables are thought to capture the information coming from a combination of SNP, genetic, and molecular markers. Latent variables can also be clustered into groups and, if relevant, such groups can be subsequently incorporated into additional latent variables. This process can be repeated to produce a hierarchical structure (a forest of latent variables) and BN analyses can be primarily completed on latent variables coupled to a largely reduced number of clinical and dosimetric features.

#### 4.3.4 Handling Missing Values

The probabilistic approach of BNs makes them suitable to efficiently handle missing values, without removal of cases or imputation. A BN can be trained even using non-complete cases and it can be queried even if a full observation of relevant features is not available. This is an advantage in clinical oncology where missing data are the norm and not the exception.

Bayesian networks were successfully applied in many oncologic/radiotherapy studies, including modeling of radiation-induced toxicity, tumor control after radiotherapy, and cancer diagnosis (169, 170, 203–207).

## 5 Improving ML Integration in Radiogenomics

Machine learning holds significant promise for advancing radiogenomics knowledge through uncovering epistatic interactions and increasing power. In this section, we will discuss general lessons learned and potential barriers.

### 5.1 Lessons From Statistics

For ML models to focus on predictive performance alone while not taking lessons from statistical theory would be a mistake. Statistical genetics learned through many iterations that it is necessary to take into account multiple hypothesis testing to decrease type I error (127). While ML models are often framed to be hypothesis-free, they can fall into a trap of cherry picking results that show good performance, which may end up being spurious. This practice of trawling for results that appear statistically significant has been called data dredging or p-hacking and has been cautioned against by the American Statistical Association (131). However, this practice can occur surreptitiously, such as when a pharmaceutical drug is tested in many highly correlated trials (i.e., asking similar questions) over many years, but without correcting for multiple testing. This phenomenon is particularly common in oncology where there is vested interest to find an application for a “blockbuster” therapeutic (208, 209). One solution for this is to create drug development portfolios to apply meta-analysis principles to drug trials instead of considering them as individuals (210). A similar approach could be used in radiogenomics to avoid publication bias and report negative results.

Notably, in their same report, the American Statistical Society emphasizes a distinction between statistical significance and clinical significance. Whether a *p*-value does or does not meet an α cutoff does not preclude it from being validated. ML provides an excellent tool for validation when used in the two-step models.

### 5.2 Reusable Hold-Out Set

Due to the nature of model building, it is often desirable to repeatedly refine one’s model due to suboptimal performance on the independent “holdout” set. Unfortunately, as discussed earlier (see Common Errors in CV), re-testing presents a significant problem as the refined model is now biased by newly obtained knowledge. For example, one might manually curate variables or alter hyperparameters to try to improve test set performance repeatedly, leading to overfitting on a true external dataset. However, reserving multiple test sets is not practical in most projects. One intriguing solution arose from university–industry collaborations with technology companies such as IBM, Microsoft, Google, and Samsung (211). These companies are interested in differential privacy, which is the concept of preserving the privacy of an individual while still collecting aggregate group statistics (212). This is not a trivial problem as knowledge about an aggregate sample over time can precisely identify supposedly “anonymous” individuals. For example, measuring the mean of a sample before and after removing one data point would allow one to precisely determine the value of that one data point if one knew the sample size. A prominent example in 2008 involved de-anonymizing publicly released Netflix data using another website (the Internet Movie Database) to ascertain apparent political affiliations and other potentially sensitive details (213). Differential privacy concepts are directly related to the necessity of maintaining independence—in essence, the “anonymity”—of the holdout set. These concepts have been adapted to a reusable holdout, whereby the holdout can be resampled many times through a separate algorithm (211, 214, 215). The number of times that the holdout can be reused grows roughly with the square of its size, thus potentially providing near-unrestricted access for large datasets such as GWAS.

### 5.3 Incorporate Clinical Variables

Many complex disease phenotypes are likely confounded by environmental effects. When genetic and environmental determinants are combined, there is increased accuracy in heritability prediction (216). This contribution from an environmental, non-genetic source suggests that multi-domain models incorporating both genetic and clinical factors should create a superior predictor compared with genetic predictors alone. Current radiotherapy prediction models focus on clinical and dosimetric variables but do not incorporate genetic factors (217). Both the ASTRO and the European Society of Radiation Oncology recognize a need for improved radiation toxicity models—including through ML (218)—and have pushed for utilization of big data toward “precision” radiation oncology (219, 220).

### 5.4 Replication and Regulatory Concerns

When applying ML to radiogenomics for eventual human applications, one must also consider practical concerns about the current regulatory environment. In the mid-late 2000s, a wave of multi-biomarker laboratory-developed tests (LDTs) in oncology emerged that made several bold, highly publicized promises. Some were met (see Precision Medicine and Multigene Panels) but many ultimately went unfulfilled. These included two proteomics-based diagnostic tests for ovarian cancer. OvaCheck (221, 222) was debunked due to data artifacts (223) and batch effects (224). OvaSure (225, 226) was pulled from market in 4 months after FDA intervention due to concerns for inadequate validation (227). Both tests reported overly optimistic positive predictive values due to being trained on unrealistic data of approximately 50% cancer positivity, whereas true ovarian cancer incidence is closer to 1 per 2,500 post-menopausal women (195–197, 227) (see Unbalanced Datasets). Certainly, the most high-profile and drawn-out case (85) involved lung cancer genomics-based chemotherapy response prediction that was pre-maturely rushed to clinical trial (228–230). Investigations into these and other controversies surrounding poor understanding of statistics and independent validation in biomarker studies (see Rashomon Effect) led to an extensive report by the Institute of Medicine which suggested corrective measures (84). Controversy continues regarding whether and how the FDA should regulate LDTs while still promoting innovation (231). One potential direction is pre-certifying laboratories instead of individual LDTs. Regardless, understanding modeling principles in a scientific environment increasingly reliant on big data analysis is necessary to avoid repeating the same mistakes of a decade ago.

### 5.5 Promoting Research

An executive summary from the ASTRO Cancer Biology/Radiation Biology Task Force (232) and a report from the ASTRO/AAPM/NCI 2016 precision medicine symposium (6) both recognized the large relative disparity between the utilization of therapeutic radiation (between 50 and 66% of cancers) and its investigative research effort. In the US, there are approximately 5,000 radiation oncologists and 15,000 medical oncologists, but a 2013 review of US National Institutes of Health (NIH) funding in radiation oncology found that <50% of all accredited departments had an active research program with at least 1 NIH grant, which is at odds with radiation oncology attracting the highest percentage of MD/PhD residents for a number of years (233). Only 3% of successfully awarded grants by the NIH Radiation Therapeutics and Biology study section are for biomarkers or radiogenomics (232). These numbers suggest that radiogenomics research continues to be underfunded. While the field moves toward improved support of young investigators through opportunities like the Holman Pathway (234, 235) and more is discovered in radiobiology and radiogenomics, there will also be a need to support methods development to ensure that radiation oncology does not lag behind in the era of precision medicine.

## 6 Conclusion

Oncology is a field enriched by multidisciplinary study. Like cancer, genetics has eluded a complete understanding due to its surprising level of complexity. The focus on ML in the technology industry is quickly moving into medicine, with a prime example being IBM Watson’s ability to understand game show questions becoming adapted for tumor board recommendations (114). These translational research efforts are not easy and require teamwork from stakeholders of varying backgrounds to avoid repeating mistakes made in one field in another field. In a radiogenomics era, radiation oncology will require multidisciplinary integration of not just radiation biologists, physicists, and oncologists but also insight from computational biologists, statistical geneticists, and ML researchers to best treat patients using precision oncology.

## Author Contributions

JK wrote the manuscript except section IV, which was written by TR, SL, and JO. SLK, JS, RS, SYK, and BR rewrote portions and made edits. All the authors approved the manuscript.

## Conflict of Interest Statement

The authors declare that the research was conducted in the absence of any commercial or financial relationships that could be construed as a potential conflict of interest.
